# Fighting cancer in coronavirus disease era: organization of work in medical oncology departments in Emilia Romagna region of Italy

**DOI:** 10.2217/fon-2020-0358

**Published:** 2020-05-21

**Authors:** Alba A Brandes, Andrea Ardizzoni, Fabrizio Artioli, Federico Cappuzzo, Luigi Cavanna, Giovanni Luca Frassineti, Antonio Frassoldati, Francesco Leonardi, Giuseppe Longo, Antonio Maestri, Davide Tassinari, Enrico Franceschi, Vincenzo Di Nunno, Carmine Pinto

**Affiliations:** ^1^Department of Medical Oncology, Azienda USL – IRCCS Institute of Neurological Sciences, Bologna, Italy; ^2^Department of Oncology, Policlinico S Orsola-Malpighi, University of Bologna, Bologna, Italy; ^3^Ospedale Ramazzini, AUSL Modena, Carpi, Italy; ^4^Department of Oncology & Hematology, AUSL Romagna, Ravenna, Italy; ^5^Department of Onco-Haematology, Guglielmo da Saliceto Hospital, Piacenza, Italy; ^6^Department of Medical Oncology, Istituto Scientifico Romagnolo per lo Studio e la Cura dei Tumori (IRST) IRCCS, Meldola, Italy; ^7^Department of Oncology, Azienda Ospedaliero Universitaria di Ferrara-Arcispedale Sant’Anna, Ferrara, Italy; ^8^Department of Oncology & Hematology, University Hospital of Parma, Parma, Italy; ^9^Oncology Division, University of Modena & Reggio Emilia, Policlinico di Modena, Modena, Italy; ^10^Medical Oncology Department, Santa Maria della Scaletta Hospital, Imola, Italy; ^11^Department of Oncology, Infermi Rimini City Hospital, Rimini, Italy; ^12^Medical Oncology Unit, Clinical Cancer Centre, AUSL-IRCCS of Reggio Emilia, Italy

**Keywords:** COVID-19, healthcare workers, medical oncology departments, oncological centresoncological patients, risk reduction, survey

## Abstract

**Aim:** To assess which and when measures were applied to reduce coronavirus disease (COVID-19) spreads have been applied in medical oncology departments. **Materials & methods:** We surveyed all medical oncology departments from the Italian Emilia Romagna region via a multidomain questionnaire. The questions covered items on patients, healthcare workers, risk reduction measure and clinical trials. **Results:** A total of 12 centers involving 861 healthcare members joined the survey. The measures applied to patients and health workers partially converged in all the departments while major divergences were found in the clinical trials domain. High rate of COVID-19 infection occurred among medical doctors (21/208, 10.1%) and social care workers (13/110, 11.8%). Rate of infection among nurses was 5.7% (24/418). **Conclusion:** All measures able to reduce risk of COVID-19 infection must be applied in medical oncology departments. Early introduction of risk reduction measures may be a critical issue.

The coronavirus disease (COVID-19) outbreak represents a world pandemic emergency [1]. The diffusion of severe acute respiratory syndrome coronavirus 2 (SARS-CoV-2) has already taken on worldwide proportions. Italy was the first European country facing significant COVID-19 infection spread. The North of Italy and in particular Lombardia region registered the higher rate of infections and COVID-19-related deaths, followed by Emilia Romagna region [2]. Italian medical centers are experiencing a significant reorganization in order to improve care for patients affected by SARS-CoV-2 and to protect healthcare workers [1,3–5].

Medical oncology departments are facing an even more complex challenge, since oncological patients seem to have a higher risk of being infected and to develop a higher rate of severe and lethal complications related to COVID-19 infection [6,7]. Therefore, every effort should converge to create a ‘COVID 19-free sanctuary’ for oncology patients. An alternative organization of clinical activity is needed to achieve this goal and preventive measures should be applied to patients, caregivers and healthcare workers [8]. National, European and American oncological-guidelines offer statements and suggestions about preferable management of patients in the COVID-19 era, however, the majority of them lacks clear instructions about the optimal organizational attitude for medical oncology departments [9]. The field experience of Italian medical oncologists resulted in the application of several precautions aimed to prevent infection within oncology departments.

Emilia Romagna region had 4,459,477 inhabitants in 2019. This region is characterized by a homogeneous distribution of specialized tertiary cancer care hospitals, with well distributed medical oncology departments that warrant no socioeconomic or geographic variations in the access to healthcare. Here, we report the results of a survey aimed to investigate the different measures applied to prevent COVID-19 infection in all 12 huge medical oncology departments from all provinces of this Italian region.

## Materials & methods

This study was designed as a multidomain survey focused on patients, healthcare workers, risk reduction measures and clinical trials in all 12 medical oncology departments from the Italian Emilia Romagna Region. The primary aim of this survey was to obtain a clear description of measures applied in medical oncology departments to prevent COVID-19 infection, as well as timing of preventive measures application and the number of people affected by COVID-19 among healthcare staff and patients. As a study population, we directed the survey to the Chiefs of all the medical oncology departments in the region (n = 12), which was completed on behalf of their departments and staff. The survey was distributed by e-mail one-time and all the Chiefs responded by e-mail within three working days to the initial query. We administered questionnaires to 12 oncological centers of the Emilia Romagna region.

The questionnaire comprised 18 questions in four domains:

- Measures applied to caregivers and patients (five questions);
- Measures applied to healthcare staff and clinical activity (seven questions);
- Measures applied to conduction of clinical trials (four questions);
- Measures applied on the management of rare or specific tumors (two questions).

Since most of medical oncology departments are reference centers for a specific tumor type, the fourth domain investigated if management of these patients has been modified during COVID-19 emergency. We collected also the number of medical doctors, nurses, social care workers and other staff members in each oncological department (including medical residents). The number of COVID-19-positive people among healthcare staff and oncological patients was also collected. We also asked to report the timing (time intervals) in which these measures have been applied according to relevant dates (first patient, ministerial decrees, restricting movement measures).

The time intervals (based on the date of release of regional or nationwide government indication) were:

- Before 22 February 2020;
- From 23 February to 5 March 2020;
- From 6 March to 11 March 2020;
- From 12 March to 21 March 2020;
- After 22 March 2020.

We also asked if some precautions (such as filtering facepiece particles [FFP] 2 or FFP3 respiratory protection masks) have been applied on the entire healthcare staff or only on more exposed healthcare workers. All questions were not necessarily mutually exclusive or completely overlapping. All healthcare workers complied with the suggested strategies in each center. There was no financial or other incentive provided to participate in this online survey. The survey results were presented with standard statistical descriptive summaries as median, percentages or quartiles.

## Results

Survey responses were obtained from all 12 medical oncology departments from the nine provinces of Emilia Romagna [10]. All these departments provide consult activity and oncological treatment administration for outpatients. Most of these departments (91.7%) have also an oncology ward.

Medical doctors, nurses and social care workers were 208, 418 and 110, respectively. Other members of the health staff (including data managers, secretaries and medical residents) were 125. Given the heterogeneous clinical activity of medical residents, we elected to include them in the ‘other’ category. Overall, 861 people composed the healthcare staff covered by the survey.

COVID-19 infection was diagnosed in 21 medical doctors (10.1%), 24 nurses (5.7%), 13 social care workers (11.8%) and one of the other members of the healthcare staff, including data managers, secretaries and medical residents (0.8%;

Figure 1).

**Figure 1.**
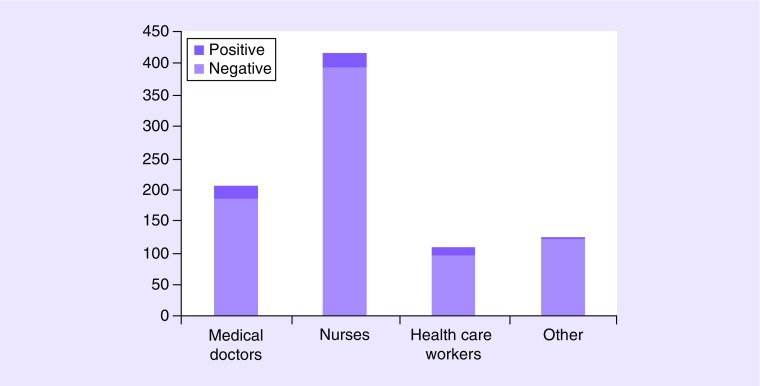
Positive and negative coronavirus disease patients among healthcare staff.

Each medical oncology department presented at least one case of COVID-19 positive patient. The overall number of patients with confirmed COVID-19 infection was 169 (range: 3–51).

## Measures applied to healthcare workers

Overall, all medical oncology departments applied some measures to protect healthcare workers from COVID-19 infection (Table 1).

**Table 1.** Measures applied (and timing of application) to reduce risk of coronavirus disease spread among healthcare staff.

Survey (number and percentage of ODs using the selected measure)		Before 22 February 2020	Between 23 February and 5 March 2020	Between 6 March and 11 March 2020	Between 12 March and 21 March 2020	After 22 March 2020	ODs adopting measures
Reduced risk of grouping		0	10 (83.3%)	2 (16.7%)	0	0	12 (100%)
Changes in work shifts		1 (8.3%)	8 (66.7%)	2 (16.7%)	0	1 (8.3%)	12 (100%)
Remote multidisciplinary meetings		0	7 (58.3%)	3 (25%)	1 (8.3%)	0	11 (91.7%)
Smart working		0	6 (50%)	5 (41.7%)	0	0	11 (91.7%)
Diagnostic swab regardless healthcare workers exposition		0	3 (25%)	0	0	0	3 (25%)
Swab in exposed members of healthcare staff		1 (8.3%)	6 (50%)	2 (16.7%)	1 (8.3%)	2 (16.7%)	12 (100%)
Assessment of antibodies		0	0	0	0	9 (75%)	9 (75%)
Adoption of PPE (all ODs)		3 (25%)	8 (66.7%)	1 (8.3%)	0	0	12 (100%)
9/12 (75%) All healthcare staff	3/12 (25%) only to exposed healthcare staff						
Surgical masks (all ODs)		3 (25%)	8 (66.7%)	1 (8.3%)	0	0	12 (100%)
12/12 (100%) All healthcare staff	0/12 (0%) only to exposed healthcare staff						
FFP2 masks (9/12 ODs: 75%)		2 (16.7%)	5 (41.7%)	0	0	2 (16.7%)	
3/12 (25%) All healthcare staff	6/12 (50%) only to exposed healthcare staff						
FFP3 masks (5/12 ODs: 41.7%)		1 (8.3%)	4 (33.3%)	0	0	0	5 (41.7%)
1/12 (8.3%) All healthcare staff	3/12 (25%) only to exposed healthcare staff						

FFP: Filtering facepiece particles; OD: Oncology department; PPE: Personal protective equipment.

All the departments applied line of work modifications and measures aimed to reduce grouping in shared spaces (rest rooms, cafeteria, locker rooms etc.). All the departments adopted personal protective equipment including gown, gloves, eye protection and surgical masks for all healthcare staff. All oncological centers carried out COVID-19 diagnostic swabs for healthcare workers exposed to patients or people with diagnosed or suspected COVID-19 infection. Most of the centers (91.7%) adopted measures aimed to promote smart-working for secretarial services and remote multidisciplinary meetings. 75% of centers adopted FFP2 masks and 25% offered these masks to all healthcare workers. Masks with higher grade of protection including FFP3 masks were adopted in 41.7% of centers, and one center (8.3%) provided these masks to all the staff. Three centers (25%) proposed diagnostic-swabs to all the staff regardless exposition to people with known COVID-19 infection, in the time interval between 23 February and 5 March 2020. Similarly, nine oncological departments (75%) offered serological test to the staff (Table 1). However, the situation is in a rapid evolution, and changes are currently ongoing.

## Changes in clinical activity

Temporary closure of oncological ward, outpatients visits or suspension of medical scheduled consults occurred in three oncological centers (25%). Follow-up visits were canceled in two centers (16.7%), delayed in seven centers (58.3%) and performed by phone or remote assessment in seven (58.3%) medical oncology departments. First oncology consults continued as usual in all oncological centers. One oncological department (8.3%) performed some consults by remote assessment and one (8.3%) centers delayed some planned primary consults. Measures applied lead to a reduction of the number of patients and treatments in three out of 12 (25%) oncological centers. Four out of 12 medical oncology departments (33.3%) prescribed oral therapies for more cycles. The number of cycle prescribed was at least two, but was dependent on treatment type, patient’s clinical conditions, disease setting. One (8.3%) center entrusted patients’ treatments to oncological center closer to their home. Overall, eight (66.7%) centers implemented the organization of blood tests close to home and oncological consult to reduce patients grouping and risk of COVID-19 exposition.

## Measures applied to patients & caregivers

All medical oncology departments minimized and/or even prohibited the presence of relatives and caregivers in the oncological department. Similarly, all centers provided a phone triage to patients one or more days before planned consult and blood tests. 11

out of 12 centers (91.7%) provided other precautions including: the adoption of surgical masks for patients or the activation of alternative care paths for patients with suspected symptoms. Eight out of 12 oncological departments (66.7%) provided diagnostic swab to patients with symptoms or clinical signs suggesting COVID-19 infection. Three out of 12 (25%) centers offered diagnostic swabs to all patients regardless the presence of symptoms. Of note, this last measure has been applied in April 2020.

## Measures applied to clinical trial & management of specific tumors in reference centers

Eight (66.7%) medical oncology departments have suspended accrual of clinical trials: this measure was limited to a number of open trials in seven centers (58.3%), and to all open trials in one (8.3%). New clinical trials were opened without reduction or with some restriction in four (33.3%) and three centers (25%), respectively. In five centers (41.7%) opening of new trails was completely discontinued. Reasons for trial restrictions were either due to sponsors or investigator choice. Nine out of 12 oncological centers (75%) are reference center for one or more tumor subtype and adopted several measures to ensure the best care even in this critical moment. These consisted on the remote management of medical treatment (50% of centers) through also remote meeting with other oncological centers (58.7%). Three oncological centers (25%) delivered oncological treatments directly to patients or to pharmacy closest to patients’ home.

## Discussion

The survey was descriptive and the aim of the study was to obtain a clear description of measures applied by front-line medical oncology departments in one of the most COVID-19 endemic areas of Italy (Emilia Romagna region). The study was not statistically powered to provide guidelines. This is the first study exploring this issue. Furthermore, our study provided important highlights about the management of COVID-19 infection in oncological centers.

Measures applied on patients, caregivers and healthcare workers seemed to converge among all oncological departments. We found only some divergences about the use of masks with higher grade of protection, the temporal interval and the execution of diagnostic swabs or serological test in patients without specific symptoms. The similarities may reflect the fast publication of joint statements from Italian Oncology Associations (AIOM -COMU - CIPOMO) [11] which provided clear instruction about the optimal management of oncological patients during COVID-19 emergency. In addition, the Federation of Italian Cooperative Oncology Groups, which brings together the 17 Italian cancer cooperative groups that have developed cancer research in Italy, in collaboration with the Italian Data Manager Group, in accordance with the communications of the Italian Medicines Agency [12], the guidelines of the EMA [13] and the recommendations of the Italian Ministry of Health [14], developed indications on the management of interventional clinical studies with drugs during the current emergency for the SARS-CoV-2 epidemic, in order to make the behavior of the clinical trial centers homogeneous, appropriate and safe [15]). Other possible explanations of some observed differences in the time of adoption of protective measures can be related to differences in the time and grade of infection spread throughout the region (ranging from 1.6 to 10.6 prevalent cases x 1000 inhabitants in the whole population), with some counties more rapidly involved than other, and to the related time of minimization procedures adopted at a general population level, based on government indications.

Of note, the role of surgical masking in the protection from COVID-19 infection is still under assessment [16]. However, the use of this personal protective equipment may reduce the likelihood of transmission from patient or healthcare workers with asymptomatic or minimally symptomatic COVID-19 infection [17–19].

Current evidence sustains the use of swab as diagnostic test while clinical symptoms and radiological imaging suggestive of COVID-19 infection could confirm the diagnosis of SARS-CoV-2 [20–22]. To our knowledge, the assessment of antibodies remains an experimental procedure. A recent study showed that seroconversion occurred in all patients affected by COVID-19: however, it was not followed by a rapid decline in viral load [23].

In our study, the management of clinical trials was heterogeneous among centers: the discrepancies across centers in the conduction of clinical trials during an outbreak reveal the lacking of clear indications on this relevant topic [12,24]. Despite measures applied, the COVID-19 infection occurred in 6.85% of the entire healthcare staff (11.8% social care workers, 10.1% medical doctors, 5.7% nurses, 0.8% other members of the staff) and in 169 patients.

## Conclusion

In our survey all medical oncology departments of Emilia Romagna region applied specific precautions on patients and healthcare staff. Nevertheless, 6.85% of health staff presented COVID-19 infection (in particular medical doctors and healthcare workers) suggesting that some specific measures and timing of measures application may be a critical issue. To date, the impact on patients’ outcomes of the above-mentioned measures applied and changes in clinical activity is unknown. Management of clinical trials in the COVID-19 era emerged as a crucial issue. Health institutions and industry have to work together to serve current trial patients to avoid lapses in care. Prioritization should be given to later stage trials with solid questions on unmet needs.

**Summary points**

- Coronavirus disease (COVID-19) represents a world pandemic emergency.
- Management of cancer patients represents an even more complex challenge in COVID-19 era.
- Measures applied to reduce COVID-19 spread should involve several domains.
- We administered a query assessing measure applied in an Italian region with high incidence of COVID-19 infection.
- Measures applied partially converged in management of patients and precautions adopted among healthcare staff.
- Divergences about management of clinical trials emerged from this survey.
- Incidence of COVID-19 infection among healthcare staff was 6.85% increasing to 10.1 and 11.8% in medical doctors and healthcare workers.
- The time in which measures have been applied could be a critical point requiring further investigation.
